# Dioctophymosis in a dog: renal and extra-renal colonization

**DOI:** 10.29374/2527-2179.bjvm007425

**Published:** 2026-03-04

**Authors:** Mariana Santos Ribeiro, Bernardo Cesar Perrone, Jussara Peters Scheffer, Adriana Fróes Barbosa, Raquel Gómez Cavalcante, Alynne da Silva Barbosa

**Affiliations:** 1 Programa de Pós-Graduação em Medicina Veterinária, Departamento de Microbiologia e Parasitologia, Instituto Biomédico, Universidade Federal Fluminese, Valonguinho, Niterói, RJ, Brazil.; 2 Veterinarian, Autonomus, Rio de Janeiro, RJ, Brazil.; 3 Departamento de Microbiologia e Parasitologia, Instituto Biomédico, Universidade Federal Fluminense. Valonguinho, Niterói, Bloco E, RJ, Brazil.

**Keywords:** Dioctophyme renale, nephrectomy, canine, helminthiasis, abdominal cavity, Dioctophyme renale, nefrectomia, canino, helmintíase, cavidade abdominal

## Abstract

This report describes the case of a young mixed-breed female dog rescued in Bahia State, Brazil, and referred for veterinary care in the city of Rio de Janeiro. Abdominal ultrasonography showed findings consistent with *Dioctophyme renale* infection in the right kidney during routine pre-surgical screening and tubular structures suggestive of free nematodes in the abdominal cavity. Right nephrectomy was performed with ovariosalpingohysterectomy after clinical, laboratory and cardiologic assessments and it resulted in the removal of eight adult helminths. The procedure was uneventful and the patient showed good postoperative recovery. This case highlights the relevance of parasitic screening in rescued animals with no prior clinical history, as well as the effectiveness of surgical intervention in unilateral dioctophymosis cases.

## Resumo

Este relato descreve o caso de uma cadela sem raça definida, jovem, resgatada no estado da Bahia e encaminhada para atendimento veterinário na cidade do Rio de Janeiro. Durante triagem pré-cirúrgica de rotina, a ultrassonografia abdominal revelou alterações compatíveis com infecção por *Dioctophyme renale* no rim direito, além da presença de estruturas tubulares sugerindo nematoides livres na cavidade abdominal. Após avaliação clínica, laboratorial e cardiológica, foi realizada nefrectomia direita associada à ovariossalpingo-histerectomia, com remoção de oito helmintos adultos. O procedimento transcorreu sem intercorrências, e a paciente apresentou boa evolução pós-operatória. O caso reforça a importância do rastreamento parasitário em animais resgatados sem histórico prévio, assim como a eficácia da abordagem cirúrgica em casos de dioctofimatose unilateral.

## Introduction

*Dioctophyme renale* is a nematode parasite of great relevance in veterinary medicine since it can cause severe damage to the kidneys of mammals, including dogs. This parasite is the largest known nematode in animals; it can reach up to 103 cm in length, in definitive hosts, such as dogs, foxes and wolves (Russo et al., 2022).

*Dioctophyme renale* life cycle involves intermediate and paratenic hosts. Initially, eggs eliminated in infected hosts’ urine develop in aquatic environments where they are ingested by aquatic annelids (intermediate hosts). Subsequently, fish predators or other paratenic hosts can become infected by ingesting these organisms. This parasite often lodges in dogs’ right kidney, but it can also be found in other locations, such as the abdominal cavity, in erratic migration cases (Russo et al., 2022).

Dioctophymosis clinical signs change depending on the renal damage. Animals are often asymptomatic at the early stages of the disease, but they can present hematuria, abdominal pain and renal function loss at advanced stages of it. Confirmatory diagnostics include urinalysis for egg identification and abdominal ultrasonography for parasitic structure visualization (Eiras et al., 2021).

Dioctophymosis cases in dogs have been mostly reported in Northern and Southeastern Brazil, but also in the Midwestern and Southern regions, at lower rates (Assis-Silva et al., 2025; Brunner et al., 2022). The hot and humid weather in the Northern region, and its large number of rivers and lakes, favor this parasite's life cycle. Studies carried out in Rio Grande do Sul State, Southern Brazil, have shown high prevalence of infection in dogs living near riverside areas, and this outcome highlights aquatic ecosystem’s role in maintaining the parasite (Brunner et al., 2022).

Variations in the geographical distribution of *Dioctophyme renale* cases in dogs across Brazil reflect environmental conditions, and cultural and socioeconomic factors. Dogs with free access to aquatic environments are at higher risk of infection, whereas animals kept in controlled conditions are rarely affected by this parasite. Accordingly, clinical case reports are essential to help identifying risk areas, since it is crucial for planning control measures, including the education of pet owners and restricted access to potential infection sources (Silveira et al., 2015). Therefore, the aim of this study was to report an *Dioctophyme renale* infection case in a dog rescued on a highway in Bahia State, Northeastern, Brazil.

## Case presentation

The patient was a mixed-breed female dog approximately two years old rescued on January 1^st^, 2025 on a highway in a rural area of Bahia State and brought to the city of Rio de Janeiro, Southeastern Brazil. It underwent routine examinations on January 10^th^, 2025 as part of a preoperative screening for an ovariosalpingohysterectomy (OSH) procedure. No clinical changes were observed on this occasion and examinations were only performed as routine check-up. Laboratory tests (complete blood count, biochemical profile and hemoparasite screening), imaging tests (abdominal ultrasonography) and cardiological assessments (echocardiogram and electrocardiogram) were carried out on the same day.

**Figure 1:**
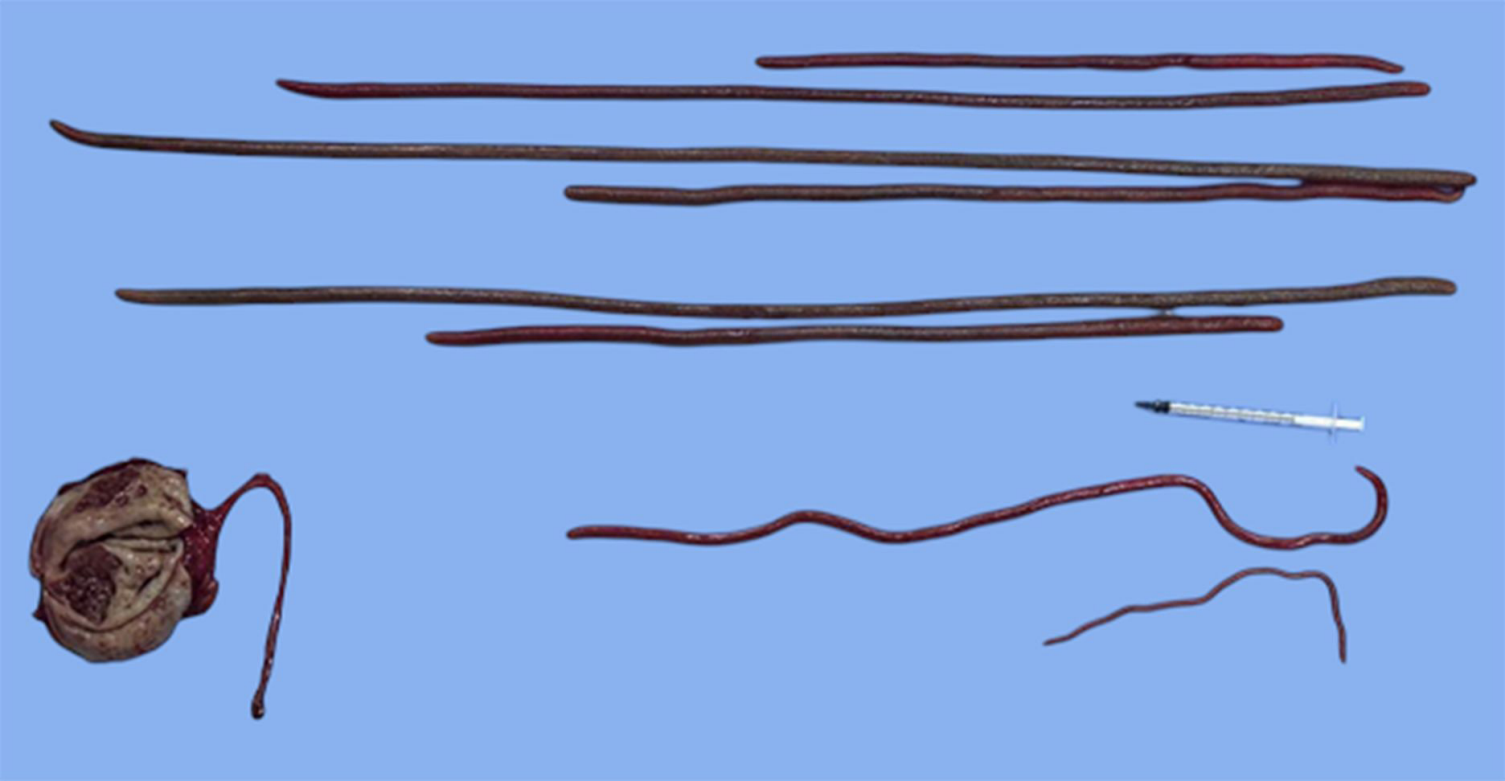
Abdominal ultrasonography showing cylindrical structures suggestive of *Dioctophyme renale* in the right kidney.

Source: Personal archive.

Abdominal ultrasonography showed the right kidney with non-featuring parenchyma presenting hyperechoic capsular structure (approximately 8.34 x 6.34 cm in size) filled with cylindrical structures (approximately 0.74 cm in diameter) suggestive of *Dioctophyme renale* nematodes. A small amount of free abdominal fluid was also observed, as well as tubular structures (up to 0.56 cm in diameter) suggestive of *Dioctophyme renale* freely dispersed in the abdominal cavity (Figure 1). Thus, the parasite was identified as incidental imaging finding, since its presence was not initially suspected. Therefore, no additional targeted diagnostic tests for parasite detection, such as urinalysis, were performed.

Hematological assessment showed mild, normocytic, normochromic anemia, with complete blood count (CBC) - 5.04 ×10^12^/L (5.5–8.5 ×10^12^/L), hemoglobin 103 g/L (120–180 g/L) and hematocrit 0.32 L/L (0.37–0.55 L/L) slightly below reference values. The reticulocyte count was 0.2% (10.08 ×10^9^/L; <60 ×10^9^/L), and it suggested non-regenerative anemia. The leukogram showed marked leukocytosis (35.32 ×10^9^/L; 5.5 –16.5 ×10^9^/L) with notable eosinophilia (15%) corresponding to 5.30 ×10^9^/L (0–1.65 ×10^9^/L), as well as absolute lymphocytosis (8.83 ×10^9^/L; 0.66–4.95 ×10^9^/L). No band neutrophils were observed ruling out a left shift. Monocyte levels (1.06 ×10^9^/L; 0–1.65 ×10^9^/L) remained within normal limits. Platelet count was 208 ×10^9^/L, within reference values (150-500 ×10^9^/L), and no thrombocytopenia, which supported the patient's hematologic stability and the planned surgical procedure safety.

Biochemical assessment showed glucose within normal limits (5.41 mmol/L; 3.33–6.11 mmol/L). Blood urea levels were mildly elevated at 9.37 mmol/L (2.5–6.67 mmol/L), whereas serum creatinine remained within reference (0.70 mg/dL; 0.50–1.50 mg/dL). Hepatic enzyme activity showed alanine aminotransferase (ALT) 36.3 U/L (17–95 U/L) and alkaline phosphatase (ALP) 55.5 U/L (10–96 U/L), both within normal limits. Total protein concentration was 67.7 g/L (60–80 g/L), with slight increase in albumin (33.4 g/L; 23–32 g/L) and normal globulin values (34.3 g/L; 27–44 g/L), which resulted in albumin:globulin ratio of 0.97 (0.5:1.1). Serum electrolytes showed sodium at 146 mmol/L (140–155 mmol/L) and potassium slightly above reference at 5.6 mmol/L (3.4–5.4 mmol/L) (Kaneko et al., 2008).

Real-time polymerase chain reaction (qPCR) assays carried out with Nova Biotecnologia® kits were performed to amplify DNA fragments targeting the following genes: *Ehrlichia canis* 16S rRNA, *Anaplasma platys* gltA, and *Babesia* sp. 18S rRNA. Two primer pairs were used for *Leishmania* spp.: one to amplify a kDNA fragment and another for quantification purposes in a separate qPCR assay by using a probe to target the 16S rRNA gene. All PCR tests were negative. Cardiologic assessments, including electrocardiogram and echocardiography, showed no abnormalities, and it allowed the patient to be safely subjected to anesthesia.

**Figure 2:**
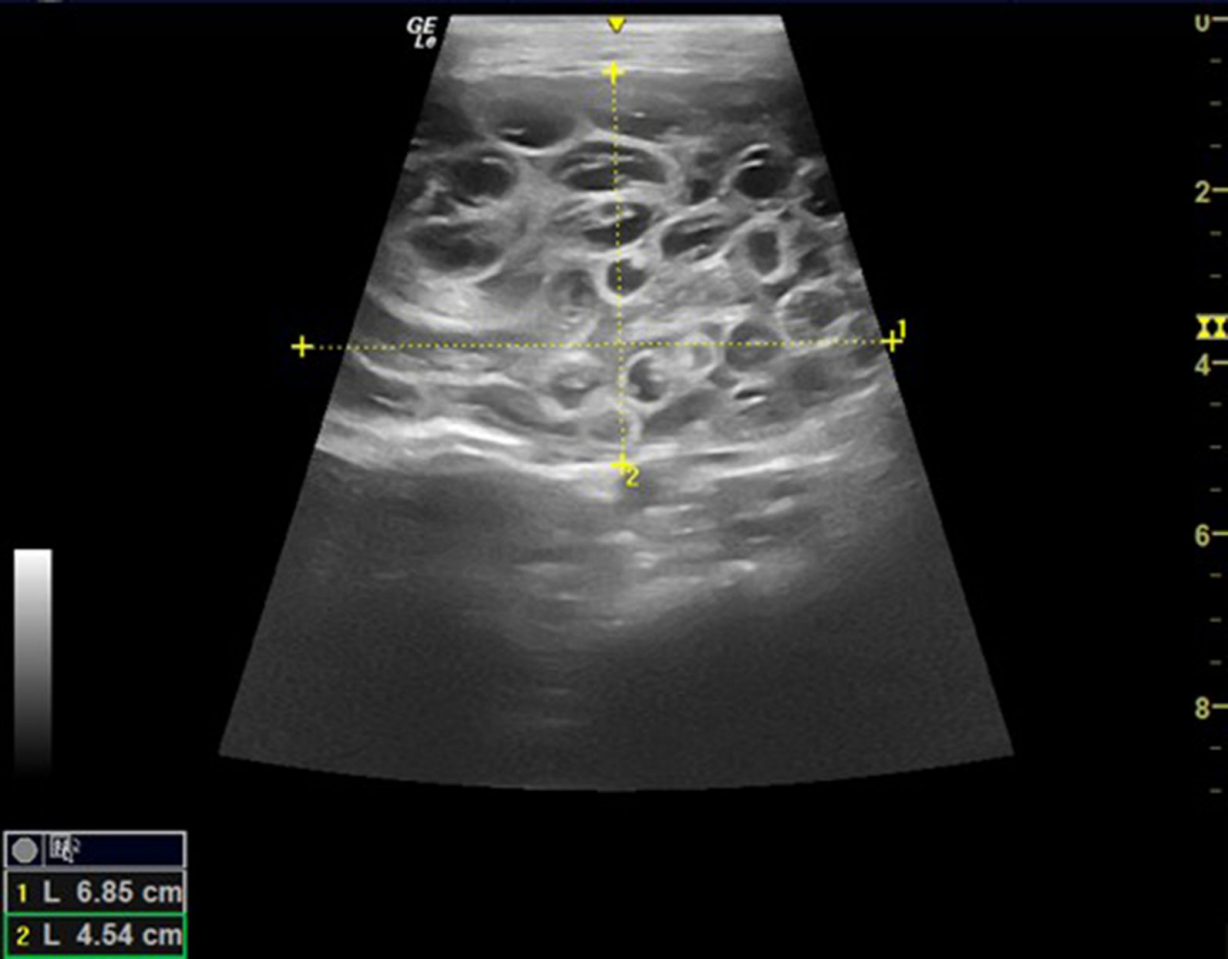
Recovered *Dioctophyme renale* specimens retrieved from kidney dissection are shown above the insulin syringe (6 cm in length); they ranged from 27.5 to 62.5 cm in size (x̄ = 45.6 ± 13.7 cm); The specimens found free in the abdominal cavity are shown below the syringe (15 and 37.5 cm, in length). The dissected renal capsule can be observed to the left of the image, and it is renal parenchyma free.

Source: Personal archive.

A right nephrectomy was performed given the diagnosis of unilateral renal dioctophymosis and the risk of complications - a thorough abdominal cavity inspection was carried out during this procedure. The procedure was performed on January 13, 2025 with vascular ligatures using poliglecaprone 2-0, prophylactic OSH with ovarian and uterine pedicle ligatures in poliglecaprone 2-0, abdominal wall closure based on Wolf pattern (poliglecaprone 2-0), subcutaneous suture through the Cushing pattern with 3-0 Vicryl and cutaneous suture through the horizontal mattress pattern with 3-0 nylon. The procedure ended without complications. The removed kidney was dissected after surgery and showed six *Dioctophyme renale* specimens ranging from 27.5 to 62.5 cm (x̄ 45.6 ± 13.7 cm) in length, as well as two additional free parasites from the abdominal cavity (15 and 37.5 cm, in length) (Figure 2).

Postoperatively, the patient was hospitalized for analgesic support and monitoring. The analgesic protocol consisted of dipyrone (25 mg/kg IV every 8 hours) and methadone (0.3 mg/kg SC every 8 hours, for 24 hours), which was later replaced by tramadol hydrochloride (4 mg/kg IV every 8 hours). Intravenous antibiotic therapy was administered with ceftriaxone (30 mg/kg every 12 hours) during hospitalization. The patient recovered from surgery with stable renal function monitored 24 hours, postoperatively.

The patient was discharged 48 hours after surgery (January 15^th,^ 2025) with the following prescriptions: dipyrone (25 mg/kg PO every 8 hours, for 5 days), tramadol hydrochloride (4 mg/kg PO every 8 hours, for 3 days) and amoxicillin-clavulanic acid (20 mg/kg PO every 12 hours, for 7 days). On January 29th, 2025, hematological and biochemical assessments were performed once more; all parameters were within the reference ranges. No parasitic structures were observed in the abdominal cavity during the postoperative follow-up ultrasound on May 5^th^ 2025. The patient has been monitored by a veterinary expert in small animal nephrology and urology. It was classified as stage 1 chronic kidney disease (CKD) based on the serum creatinine levels. According to the International Renal Interest Society (2023) guidelines, the condition is further subclassified as normotensive and non-proteinuric.

## Discussion

The patient in this case report was incidentally diagnosed during preoperative screening for an OSH procedure. This scenario highlighted how imaging examinations are underscored, even for apparently healthy animals, mainly in semi-stray or stray dogs from endemic regions. Dioctophymosis is a helminthiasis of low overall prevalence (~1%) but it is considered endemic in certain Brazilian regions, mainly in areas with access to contaminated waterbodies (Assis-Silva et al., 2025).

*Dioctophyme renale*, known as giant kidney worm, is a parasite showing renal tropism. It causes progressive renal parenchyma destruction since it often remains asymptomatic for long periods-of-time (Russo et al. 2022; Leite et al., 2005). However, extra-renal locations can be affected in up to 60% of cases and the asymptomatic nature of many infections makes diagnosis more complicated (Acha & Szyfres, 2003). These findings are in compliance with the present case, according to which, two free parasites were found in the abdominal cavity. Ultrasonography played decisive role in identifying both the renal damage and the unexpected presence of free parasites in the abdominal cavity. The presence of helminths outside the urinary tract can be related to renal capsule rupture secondary to chronic tissue necrosis or active parasite migration (Verocai et al., 2009; Greer et al., 2021).

Although the patient showed no clinical signs of this parasitosis, CBC was consistent with a chronic parasitic condition; it showed non-regenerative normocytic normochromic anemia compatible with chronic inflammatory or with systemic diseases (at slower progression) such as chronic tissue parasitic infections (Weiss & Wardrop, 2010). In addition, marked eosinophilia and absolute lymphocytosis were observed, and it reflected immune response to prolonged tissue infection. Intense eosinophilia is compatible with parasitic infections, mainly when it is caused by large helminths like *Dioctophyme renale*, which points toward active immune response. Lymphocytosis, in its turn, suggests chronic antigenic stimulation (Kritsepi‑Konstantinou & Oikonomidis, 2016).

Lack of significant changes in biochemical and cardiologic tests opened room for a safe surgical approach, under anesthesia. Furthermore, the ruling out of co-infections caused by hemoparasites improved the predictability of surgical prognosis. Nephrectomy was justified by complete renal parenchyma destruction and risk of peritonitis and parasitic dissemination. OSH was a preventive measure, mainly in a patient with no reproductive interest; it reduced the need for future anesthetic-surgical interventions. This approach is in compliance with the best surgical practice guidelines for females without breeding value (Romagnoli et al., 2024).

The postoperative recovery was satisfactory, with appropriate pain management and the preservation of the remaining renal function. The patients’ current classification as IRIS stage 1 CKD, normotensive and non-proteinuric (IRIS, 2023) highlights the favorable prognosis when early diagnosis and timely intervention are achieved.

## Conclusion

This case highlights the importance of screening examinations for *Dioctophyme renale* detection in dogs with unknown clinical history, mainly in the rescued or adopted ones. Abdominal ultrasonography proved essential for diagnosis since it allowed visualizing renal and extra-renal infection even in the absence of clinical signs or biochemical changes in renal markers. The patient was classified as stage 1 chronic kidney disease based on imaging findings and surgical intervention. Early identification is crucial for continuous monitoring and for the implementation of measures aimed at ensuring the patient’s quality of life and longevity.
